# Integrating Screens and Spoons: An Exploratory Study on Digital Technology’s Influence on Parent–Child Interactions

**DOI:** 10.3390/ejihpe15030036

**Published:** 2025-03-16

**Authors:** Silvia Cimino, Luca Cerniglia

**Affiliations:** 1 Department of Dynamic, Clinical and Health Psychology, Sapienza University of Rome, 00185 Roma, Italy; 2 Faculty of Psychology, International Telematic University Uninettuno, 00186 Roma, Italy; luca.cerniglia@uninettunouniversity.net

**Keywords:** child development, parent–child interaction, digital devices, family routines

## Abstract

Background: Parent–child interactions during mealtime significantly influence social, emotional, and cognitive development in early childhood. Increasing parental use of digital technology has been linked to disruptions in these interactions, a phenomenon termed “technoference,” which is associated with relational conflicts and psychosocial difficulties in children. Feeding interactions are particularly important for fostering attachment and emotional regulation, making them a vital area for studying technology’s effects on parent–child dynamics. Aims: This study aimed to evaluate the impact of parental digital technology use during mealtime on the quality of feeding interactions and child dysregulation symptoms. Two groups were compared: mothers who used devices during mealtime (Technology Group, TG) and mothers who did not (Non-Technology Group, NTG). Methods: Participants included mother–child dyads (TG) and a matched control group (NTG) (Ntot = 174), selected from a broader research project. Mother–child pairs were observed during a 20 min midday meal using the validated Italian Scala di Valutazione dell’Interazione Alimentare (SVIA). The emotional and behavioral functioning of children was assessed with the Child Behavior Checklist (CBCL). Statistical analyses included ANOVAs and post hoc tests. Results: The TG group demonstrated significantly higher scores on all SVIA subscales, indicating greater parental affective challenges, conflict, food refusal behaviors, and dyadic distress. Additionally, children in the TG group exhibited more internalizing and externalizing problems, including dysregulation symptoms on the CBCL, compared to the NTG group. Conclusions: Parental technology use during mealtime negatively affects parent–child feeding interactions and increases dysregulation in children. These findings highlight the need for mindful parenting strategies to limit digital distractions and foster healthier family dynamics.

## 1. Introduction

### 1.1. Parent–Child Interactions and Child Development

Exploring the intricacies of parent–child mealtime interactions during early childhood, particularly in the context of increasing digital technology use, is crucial for understanding developmental challenges (Brauchli et al., 2023; Paulus et al., 2024). These interactions play a pivotal role in shaping a child’s social, emotional, and cognitive development (Nguyen et al., 2024; Liu et al., 2024). Research shows that intentional, focused engagement between parents and children can mitigate the negative effects of excessive screen time, reducing relational conflicts and improving children’s executive functioning such as emotion regulation, cognitive flexibility, and problem solving (Sadeghi et al., 2019). Conversely, increased technology use by parents is often linked to reduced engagement with their children, leading to more screen time for children and contributing to psychosocial issues, especially in families with lower socioeconomic status (Wong et al., 2020).

Early parental interactions are essential for fostering autonomy and developing self-regulation in young children (Bernier et al., 2010). Parent–infant synchrony, which supports self-regulation, empathy, and symbolic understanding, can be disrupted by technology use (Feldman, 2007). Early adult–infant interactions are believed to significantly impact an individual’s long-term adjustment and psychopathology development (Salcuni & Simonelli, 2018). In particular, feeding interactions are vital for achieving developmental milestones like attachment and emotional regulation (Satter, 1990). Additionally, technology use during family meals can reduce perceptions of family closeness (Nelson, 2019), with mobile phone use yielding both positive and negative effects on family communication, highlighting the complex role of technology in shaping family dynamics (Elias et al., 2021).

### 1.2. Technology Use and Its Relations to Parent–Child Interactions

The concept of “technoference”—the disruption of family interactions due to technology use—has been examined in relation to child behavior problems, highlighting the complex interplay between technology and family dynamics (Swider-Cios et al., 2023). Research on parental preferences for mealtime technology suggests a stronger inclination toward screen-based devices over voice interfaces and smart objects, primarily due to concerns about distraction and over-reliance on technology (Pereira-Castro et al., 2022).

Studies analyzing family interaction patterns have identified significant correlations between these dynamics and children’s externalizing and internalizing problems, underscoring the reciprocal influence of relational and individual factors in the development of psychopathology (Cimino et al., 2016; Gatta et al., 2017; Minuchin, 1974; Cerniglia et al., 2020). The responses of parents to children with specific needs, such as those with cerebral palsy during feeding, have been scrutinized, highlighting the necessity of tailoring interactions to the unique needs of the child (Ballarotto et al., 2021; Olrick et al., 2002).

The establishment of parent–infant interactions and their influence on developmental outcomes have been discussed, with dysregulation linked to problematic behaviors and developmental impairments (e.g., ADHD and/or defiant–oppositional behavior) (Evans et al., 2020). Observations of infants’ responses to a mobile phone-modified still-face paradigm have shown increased negative affect and escape behaviors during phases when the parent is distracted by the phone, underscoring the impact of parental distraction by technology on infant behavior (Stockdale et al., 2020).

The relationship between technological interruptions in parent–child interactions and child behavior problems has been further investigated, suggesting that disrupted or less than optimal interactions can have detrimental effects on child development (Elias et al., 2021). The interaction patterns of infants referred to mental health clinics have been studied, revealing less optimal mother–infant interactions and environments compared to non-referred pairs (Keren et al., 2001). Observational methods to evaluate mother–child mealtime interactions established a link between parental feeding practices and the child’s weight status (Bergmeier et al., 2015; Cimino et al., 2020).

This exploratory study aimed to verify whether the use of digital technologies during feeding interactions has an influence on the quality of these exchanges. In particular, this research intended to assess the quality of feeding interactions and possible dysregulation symptoms in children by comparing two groups. One group was composed by dyads in which mothers used digital technologies (smartphones and/or tablets) during mealtime (Technology Group—TG). The other group, paired for socio-anamnestic characteristics, was composed by dyads in which mothers did not usually use digital technologies (smartphones and/or tablets) during mealtime (Technology Group—NTG).

## 2. Methods

### 2.1. Sample

Mothers and children participating in this study were selected from a larger research (N_tot_ = 412) project aimed at promoting the psychological well-being of offspring within the general population (Cimino et al., 2016). The broader study included the administration of anamnestic questionnaires that addressed, among other topics, the use of technology such as smartphones and iPads. From this dataset, a subgroup of mothers who reported using such devices with their children (TG; N = 89) was identified by the authors of this study, while a control group (NTG; N = 85) was exactly matched based on maternal age and socio-economic status (SES). The average age of mothers was 29.32 years (SD = 4.03), and the children’s average age was 2.53 years (SD = 1.04), with 52% being female. All mothers were biological parents, primary caregivers, and in daily contact with their children. The majority of families (95%) were two-parent households, all participants were Caucasian, and the group exhibited a middle-to-high SES, with household incomes ranging between €35,000–€45,000 annually. The sample was predominantly drawn from urban and suburban areas. No significant differences were found between the TG and NTG groups on these characteristics (χ²; *p* < 0.001). Exclusion criteria included psychiatric diagnoses or ongoing medical/psychological treatment. All mothers provided written informed consent, with ethical approval granted before the study commenced (N. 13/2022).

Mother–child pairs were observed at home during 20 min video recordings of a midday meal. These sessions were conducted by psychologists trained in using an observational tool. Two independent raters, also trained, coded the videos using a standardized manual (Stockdale et al., 2020) and employed both paper–pencil systems and specialized software to analyze subscale scores. The observation followed a validated Italian protocol, the Scala di Valutazione dell’Interazione Alimentare (SVIA), or the Observational Scale for Mother–Infant Interaction during Feeding (Lucarelli et al., 2003), which has been extensively applied in prior research. This protocol was specifically chosen for its focus on feeding interactions, setting it apart from other observational methods.

### 2.2. Measures

#### 2.2.1. Parent–Child Feeding Interactions

The Italian SVIA Feeding Scale (validated for Italian population; (Lucarelli et al., 2003)) includes 41 items rated on a Likert scale from 0 (none) to 3 (many), organized into four subscales that separately evaluate mother–child and father–child interactions. The subscales assess: Affective State of the Parent: higher scores indicate difficulties in expressing positive affect and frequent displays of sadness or distress. Interactional Conflict: evaluates the degree and intensity of conflict within the dyad. Food Refusal Behaviors by the Child: examines emotional and behavioral traits related to feeding patterns. Affective State of the Dyad: higher scores reflect challenges in supporting the child’s autonomy, often accompanied by distress or oppositional behavior. The Italian SVIA demonstrates strong discriminant validity (82–92% correct classification) and interrater reliability (0.82–0.92).

#### 2.2.2. Children’s Emotional–Behavioral Functioning

The Child Behavior Checklist (CBCL; version validated for Italian population (Achenbach et al., 2017)) evaluates emotional–behavioral functioning in children aged 1½ to 5 years. Parents rate 99 problem items on a scale from 0 (not true) to 2 (very true) based on the preceding two months. Problem behaviors are grouped into six syndromes across two scales: internalizing problems (e.g., emotionally reactive, withdrawn) and externalizing problems (e.g., attention issues, aggression). The CBCL dysregulation profile (DP) combines scores for anxious/depressed, attention problems, and aggressive behavior. The reliability (Cronbach’s alpha) for this measure is 0.93.

### 2.3. Data Analysis

Initial data screening identified 3% missing data per instrument, handled using multiple imputations in SPSS (Version 25.0). ANOVAs assessed interaction patterns between TG and NTG dyads during feeding using SVIA dimensions and CBCL subscales. Univariate analyses followed significant results, with post hoc Bonferroni-adjusted Duncan tests. Child gender and maternal age did not significantly affect variables across analyses. Statistical tests were performed in SPSS (Version 25.0).

## 3. Results

### Quality of Mother–Child Feeding Interactions

ANOVA revealed significantly higher scores for the TG group compared to NTG across all SVIA subscales (*p* < 0.01). Approximately 11% of TG dyads exceeded clinical cut-offs (>54) for maladaptive interactions on the SVIA. Time-based effects were significant across all SVIA subscales (*p* < 0.001), with post hoc tests showing lower maladaptive behaviors in NTG dyads. Subscale analyses indicated that TG dyads had higher scores for parental affective difficulties, interactional conflict, food refusal behaviors, and negative emotional states within the dyad. Table 1 presents subscale means and eta² values for both groups, highlighting effect sizes.

Mothers in NTG also rated their children’s emotional and behavioral functioning as less maladaptive, particularly on the withdrawn, anxious/depressed, and aggressive behavior subscales. Additionally, children in NTG exhibited significantly lower scores on both the internalizing and externalizing problem scales, as well as the dysregulation profile scores. Table 2 presents the means and eta^2^ values.

## 4. Discussion

This study explored the influence of maternal digital technology use during mealtime on the quality of mother–child feeding interactions and children’s emotional–behavioral functioning. The findings highlight significant differences between the Technology Group (TG) and the Non-Technology Group (NTG) across multiple dimensions of feeding interaction quality and child behavior outcomes. These results underscore the complex role of technology in shaping early parent–child dynamics, with both strengths and limitations to consider.

The TG demonstrated significantly higher scores across all SVIA subscales, indicating poorer interaction quality, including more parental affective difficulties, higher levels of conflict, and increased food refusal behaviors. These findings align with previous research indicating that maternal technology use during interactions can disrupt parent–child synchrony and decrease parental responsiveness (Wolfers et al., 2020). This disruption may impair the development of emotional regulation and autonomy in children, as suggested by the higher levels of maladaptive interactions observed in TG dyads.

On the other hand, NTG dyads exhibited fewer maladaptive behaviors, which aligns with literature emphasizing the importance of focused and distraction-free parent–child interactions during mealtime for fostering positive developmental outcomes (Choy et al., 2024). The lower scores for NTG dyads on measures of conflict and negative affective states suggest that uninterrupted engagement can enhance the emotional connection between parent and child.

Children in the TG exhibited higher internalizing and externalizing problem behaviors as well as more severe dysregulation profiles (DPs) compared to those in the NTG. These results are consistent with studies linking increased parental technology use to heightened child behavior problems, likely mediated by decreased parental sensitivity and increased negative parent–child interactions (Glassman et al., 2024).

Notably, the lower levels of internalizing and externalizing behaviors observed in the NTG are supported by evidence that intentional and engaged parenting can buffer children from emotional and behavioral maladjustments (Lee & Chae, 2012). By contrast, technoference appears to disrupt the protective effects of sensitive caregiving, as reflected in the heightened levels of maladaptive behaviors in the TG.

The findings of this study corroborate earlier work highlighting the disruptive role of parental technology use during key developmental interactions (Sun et al., 2024).

However, some studies suggest that the effects of parental technology use may depend on contextual factors, such as the content of technology use or the nature of the parent–child relationship (Liszkai-Peres et al., 2024). For example, educational technology or the co-viewing of media may yield less detrimental effects than solitary technology use, which was not explored in this study.

Contradicting findings from a few studies that highlight potential benefits of digital technology, such as facilitating shared activities or providing opportunities for co-regulation (Shao et al., 2024), this study’s results suggest that solitary maternal technology use during feeding interactions is predominantly associated with negative outcomes. These differences may stem from variations in study designs, measurement tools, and sample characteristics.

A key strength of this study is its use of validated, standardized observational measures, such as the SVIA and CBCL, to assess both interaction quality and child outcomes. The inclusion of a matched control group further enhances the validity of the comparisons, ensuring that differences between groups are not attributable to socioeconomic or demographic confounds. Additionally, the use of in-home observations provides ecological validity, capturing feeding interactions in a naturalistic setting rather than a laboratory context. However, several limitations must be considered. First, the cross-sectional design precludes causal inferences about the relationship between maternal technology use and child outcomes. While significant associations were identified, the directionality of these relationships remains unclear. For instance, it is possible that mothers of children with pre-existing behavioral challenges are more likely to engage in technology use during mealtime as a coping mechanism (Sharma et al., 2024).

Second, the sample’s homogeneity in terms of socioeconomic status (middle-to-high SES) and ethnicity limits the generalizability of the findings to more diverse populations. Research has shown that the effects of technoference may be exacerbated in families with lower SES or greater stress levels (Ludlow et al., 2024). Future studies should include more diverse samples to explore the potential moderating effects of socioeconomic and cultural factors. Third, this study did not examine the impact of different types of technology use (e.g., educational apps versus social media) and did not discuss possible interventions that can mitigate the effects of technology.

Finally, the reliance on self-reported technology use and behavioral ratings may introduce bias. Although observational methods were employed for feeding interactions, future studies could benefit from objective measures of technology use (e.g., screen-time tracking apps) to validate self-reports and reduce potential inaccuracies.

The findings emphasize the importance of minimizing distractions during parent–child mealtime interactions to promote better relational and developmental outcomes. Interventions aimed at reducing parental technology use during mealtime could be particularly beneficial, especially in families with young children. Additionally, future research should examine longitudinal effects to better understand how early feeding interactions influenced by technoference impact developmental trajectories over time.

Moreover, exploring potential moderating variables, such as parental stress levels, child temperament, and cultural norms, could provide a more nuanced understanding of how and why technology use impacts parent–child dynamics. Finally, integrating objective measures of technology use and real-time assessments of interaction quality could address some of the methodological limitations of the present study.

## 5. Conclusions

This study highlights significant differences in mother–child feeding interactions and child emotional–behavioral functioning associated with maternal technology use during mealtime. While the findings align with prior research indicating negative effects of technoference, the study’s cross-sectional design and sample homogeneity necessitate caution in generalizing these results. Further research is needed to explore the causal mechanisms underlying these associations and identify effective strategies for mitigating the impact of parental technology use on child development.

## Figures and Tables

**Table 1:** Mean scores and standard deviations of the SVIA subscales and general quality of mother–child feeding interactions.

	TG	NTG	η^2^
	M (SD)	M (SD)	
Mother’s affective state	21.14 (2.45)	15.62 (1.53)	0.67 **
Interactive conflict	16.14 (2.11)	14.45 (2.35)	0.68 **
Food refusal behavior	18.36 (2.46)	14.74 (2.54)	0.64 **
Dyad’s affective state	21.26 (1.55)	16.46 (2.32)	0.61 **
General quality	50.81 (1.52)	34.6 7(2.52)	0.76 **

Note. ** *p* < 0.001. η^2^: eta-squared.

**Table 2:** Mean scores and standard deviation of the child’s CBCL subscales.

	TG	NTG	η^2^
INT	18.47 (1.71)	14.73 (1.53)	0.61 **
EXT	13.64 (2.35)	10.31 (1.65)	0.58 **
DP	13.67 (1.89)	11.83 (2.44)	0.72 **

Note. INT: internalizing problems; EXT: externalizing problems; DP: dysregulation profile. η^2^: eta-squared. ** *p* < 0.001.

## Data Availability

Dataset available on request from the authors.
